# Diagnostic and Therapeutic Impact of FDG‐PET/CT Following MRI Staging in Anal Cancer: A Systematic Review and Meta‐Analysis

**DOI:** 10.1111/1754-9485.70071

**Published:** 2026-01-19

**Authors:** Hugo C. Temperley, Jack M. Bell, Avinash Deshwal, Wanyang Qian, Tom O. Cuddihy, Benjamin M. Mac Curtain, Niall J. O'Sullivan, Kevin P. Sheahan, Paul H. McCormick, Alison Corr, Niall Sheehy, James F. M. Meaney, Michael E. Kelly

**Affiliations:** ^1^ Department of Radiology St James's Hospital Dublin Ireland; ^2^ Trinity College Dublin St James's Cancer Institute Dublin Ireland; ^3^ Department of Radiology Beaumont Hospital Dublin Ireland; ^4^ Royal College of Surgeons in Ireland (RCSI) Dublin 2 Ireland; ^5^ Department of Surgery St John of God Subiaco Hospital Subiaco Western Australia Australia; ^6^ Northwell Health New Hyde Park NY USA; ^7^ Department of Urology Lenox Hill Hospital New York NY USA; ^8^ Department of Colorectal Surgery St James's Hospital Dublin Ireland; ^9^ The Thomas Mitchell Centre for Advanced Medical Imaging (CAMI), St. James' Hospital Dublin Ireland

**Keywords:** anal cancer, diagnostic accuracy, MRI, positron emission tomography, TNM staging

## Abstract

**Introduction:**

Accurate staging is essential in anal cancer to guide therapy and prognostication. While MRI remains the modality of choice for local staging, its limitations in assessing nodal and distant metastases have prompted evaluation of FDG PET/CT as an adjunct. The American College of Radiology recommends FDG‐PET/CT as a complementary modality for initial staging, particularly for nodal assessment.

**Methods:**

A systematic search of PubMed, EMBASE, Web of Science and Scopus was conducted up to August 2025 following PRISMA guidelines (PROSPERO ID: CRD1149778). Studies included reported adult patients with biopsy‐proven anal squamous cell carcinoma who underwent both MRI and FDG‐PET/CT for initial staging. Primary outcomes included per‐patient sensitivity/specificity for metastasis, changes in TNM staging and therapeutic outcomes, including management modification.

**Results:**

Six studies (*n* = 246) met the inclusion criteria. Five studies reported on staging changes, where FDG‐PET/CT altered staging in 22.5% (95% CI: 12.3–34.7) of patients, more commonly through upstaging than downstaging (16.2% [95% CI: 10.7–22.5] vs. 6.3% [95% CI: 1.5–14.2]). Upstaged patients were predominantly nodal (74.6% [95% CI: 63.2–83.1]). Previously occult metastases were identified with FDG PET/CT in 3% (95% CI: 1.1–6.9) of patients. Management changes occurred in 20.7% (95% CI: 14.9–27.4), predominantly through radiotherapy field expansion or dose modifications.

**Conclusion:**

FDG‐PET/CT following MRI provides incremental diagnostic and therapeutic value in anal cancer staging, through refining nodal and metastatic staging and influencing radiotherapy planning, supporting its routine integration to optimise staging accuracy and management decisions.

**Trial Registration:**

PROSPERO: CRD42023446290

## 1 Introduction

Anal squamous cell carcinoma (ASCC) is a relatively uncommon malignancy, accounting for 2% of gastrointestinal cancers. Still, its incidence is increasing worldwide, particularly among populations with human papillomavirus (HPV) infection and immunosuppression [1, 2]. Accurate baseline staging is critical for prognosticating and defining optimal radiotherapy (RT) target volumes.

Pelvic magnetic resonance imaging (MRI) is the reference standard for local staging owing to its superior soft‐tissue contrast and ability to assess tumour extent, sphincter involvement and mesorectal fascia integrity [3]. However, MRI relies on morphological criteria, such as nodal size and contour, which limit its sensitivity for micrometastatic disease and distant spread. Because nodal status and distant metastases directly influence prognosis and RT planning, comprehensive systemic evaluation remains essential.

18F‐fluorodeoxyglucose positron emission tomography/computed tomography (FDG‐PET/CT) integrates metabolic and anatomic data, detecting hypermetabolic lesions that may be occult on MRI. Multiple studies demonstrate that PET/CT enhances detection of nodal and distant metastases, frequently altering TNM stage or management [4, 5, 6]. These data underpin recommendations from the American College of Radiology (ACR) and the ESMO–ESSO–ESTRO guidelines, which endorse PET/CT as a complementary modality for baseline staging and RT planning [3, 7].

Yet, the incremental benefit of FDG‐PET/CT after high‐quality MRI remains uncertain. Earlier meta‐analyses often included heterogeneous imaging protocols, limiting relevance to current MRI‐based workflows [8, 9]. Recent evidence indicates that even after MRI, PET/CT can reveal previously occult nodal or metastatic disease, particularly in inguinal, iliac and para‐aortic regions, prompting RT modification. At the same time, metabolically inactive nodes may be reclassified as benign [10, 11]. PET‐derived staging also correlates more strongly with survival outcomes [12].

This systematic review and meta‐analysis quantifies the diagnostic and therapeutic impact of FDG‐PET/CT following MRI staging in ASCC, providing an updated assessment of its residual value within modern multimodality pathways.

## 2 Methods

### 2.1 Study Design and Reporting Guidelines

This study is a systematic review and meta‐analysis of randomised and non‐randomised studies evaluating the diagnostic and therapeutic impact of FDG‐PET/CT following MRI staging in anal cancer. The review was conducted in accordance with the Preferred Reporting Items for Systematic Reviews and Meta‐Analyses (PRISMA) guidelines.

### 2.2 Search Strategy

A comprehensive literature search was performed in the following databases: Medline (via PubMed), EMBASE, Web of Science and Scopus. Searches were conducted up to 25/08/25. Detailed search strategies for each database are provided in Appendix S1. Grey literature sources (including Google Scholar and reference lists of relevant articles) were also screened to capture ongoing or unpublished studies. Only studies published in English were included.

(‘Anal Canal’[Mesh] OR anal cancer OR anal carcinoma OR ‘anal squamous’) AND (PET CT OR PET/CT OR ‘Positron‐Emission Tomography Computed Tomography’ OR FDG OR fluorodeoxyglucose) AND (MRI OR MR imaging OR ‘Magnetic Resonance Imaging’[Mesh]) AND (staging OR stage OR upstage* OR downstage* OR nodal OR metastases OR management OR radiotherapy OR treatment plan*).

### 2.3 Inclusion Criteria

Studies were eligible if they met the following criteria:

Studies that satisfied the inclusion and exclusion criteria were included. The following PICO elements were used as the basis for selecting studies:

- Population (P): Adults with biopsy‐proven ASCC undergoing initial staging MRI.
- Index test (I/E): FDG‐PET/CT performed after MRI (within the primary staging pathway).
- Comparator (C): Conventional staging without PET/CT (MRI ± CT/US/EUS), or MRI alone.
- Outcomes (O):
  ○ Diagnostic: Per‐patient sensitivity/specificity for nodal and distant metastases; change in TNM stage (up/down).
  ○ Therapeutic: Management change attributable to PET/CT (e.g., RT field/boost modification, change in intent).

### 2.4 Exclusion Criteria

- Case reports, case series (< 10 patients), reviews, conference abstracts, editorials.
- Patients with cancers other than ASCC.
- Patients without MRI staging before PET/CT
- Studies evaluating PET/CT only
- Qualitative or narrative data without extractable diagnostic or therapeutic outcomes.

### 2.5 Study Selection, Data Extraction & Critical Appraisal

A database was created using the reference management software EndNote X9 TM. Two researchers (H.C.T. and B.M.M.C.) independently reviewed the search outputs. Initially, duplicates were removed. Study titles were then screened and assessed for potential relevance. The abstracts of selected potential studies were then read and evaluated for eligibility for inclusion according to the inclusion/exclusion criteria detailed above. Rejected studies were grouped in the database by their reason for exclusion. The full texts of the abstracts deemed eligible for inclusion were then further analysed using the same criteria. Conflicts between the two reviewers (H.C.T. and B.M.M.C.) were resolved following an open discussion and final decision by the senior author (M.E.K.).

In order to extract and store data efficiently, the Cochrane Collaboration screening and data extraction tool, Covidence, was used [13]. Data was collected by two reviewers (N.O.S. and H.C.T.) independently, using the following headings: study details, study design, population, intervention, comparison groups and outcomes. Basic participant characteristics were also extracted. Conflicts between the two reviewers were resolved following an open discussion and final decision by the senior author (M.E.K.).

A critical appraisal of the methodological quality and risk of bias of the included studies was performed. The critical appraisal was completed independently by two reviewers. Quality assessment of non‐randomised controlled trials (non‐RCTs) was performed according to Newcastle–Ottawa Scale (NOS) [14]. We assigned stars to evaluate study quality: 7 stars ‐ ‘very good’, 5–6 stars ‐ ‘good’, 3–4 stars ‐ ‘satisfactory’ and 0–2 stars ‐ ‘unsatisfactory’. The critical appraisal was completed by two reviewers independently (H.C.T. and B.M.M.C.). Quality assessment of randomised‐controlled trials was performed using the Cochrane Collaboration risk of bias tool [15].

### 2.6 Data Analysis

Statistical analysis was performed using Revman Statistical Software (Ver. 5, Copenhagen, Denmark) [16]. Binary outcome data were reported as odds ratio (OR) and 95% confidence interval (95% CIs) were estimated using the Mantel–Haenszel method. For continuous data, mean differences and 95% CI were estimated using inverse variance weighting. Outcome measures (mean + standard deviation (SD) and median + interquartile range (IQR)) were recorded. If needed, outcome variables (mean and SD) were estimated from the median and range using formula described by Hozo et al. [17]. Heterogeneity was assessed by *I* ^2^ statistics, with > 50% being considered as considerable heterogeneity. Random effects modelling was applied in cases of increased heterogeneity (> 50%) and a fixed effects model applied otherwise. Statistical significance was attributed to *p*‐value < 0.05.

### 2.7 Systematic Review Registration

Our systematic review was registered on PROSPERO in August 2025.

## 3 Results

### 3.1 Literature Search

**FIGURE 1:**
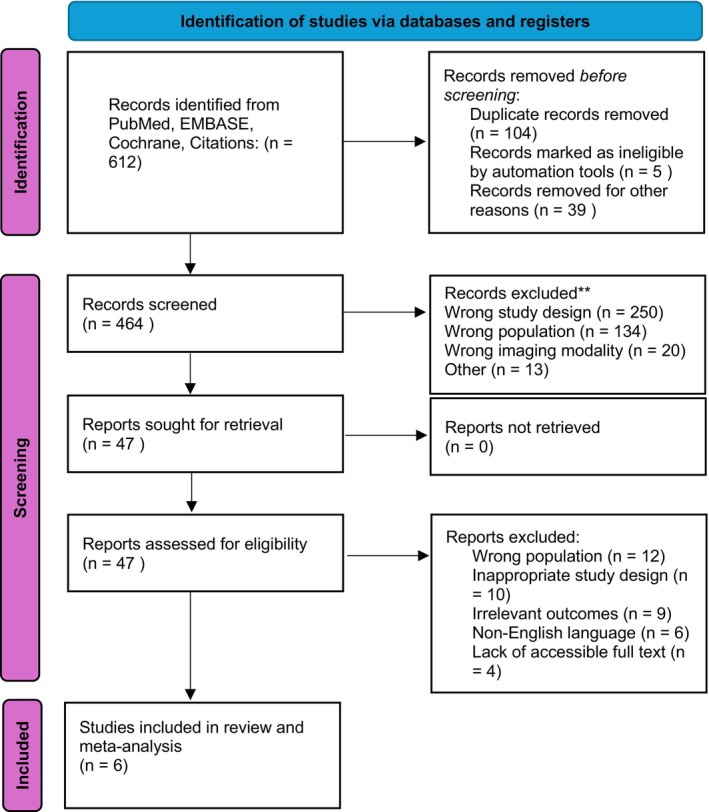
PRISMA Statement for Impact of FDG‐PET/CT following MRI staging in anal cancer. *Source:* Page et al. [21].

A systematic search of PubMed, EMBASE and Cochrane, supplemented by citation snowballing, yielded 612 records. After removal of duplicates (*n* = 148), 464 records were screened by title and abstract. Of these, 47 full texts were retrieved for detailed review. A total of 41 studies were excluded for reasons including wrong population (*n* = 12), inappropriate study design (*n* = 10), irrelevant outcomes (*n* = 9), non‐English language (*n* = 6) or lack of accessible full text (*n* = 4). Six studies fulfilled the predefined eligibility criteria and were included in the final systematic review and meta‐analysis (Bhuva [18], Di Carlo [5], Engledow [19], Horvat [4], Manafi‐Farid [6], Wells [20]). See Figure 1.

### 3.2 Study Characteristics

Six studies, published between 2011 and 2024, were included. Four were retrospective single‐centre series (Bhuva [18], Di Carlo [5], Horvat [4], Manafi‐Farid [6]), while two were multicentre (Engledow [19], Wells [20]), including one prospective study (Engledow [19]). Sample sizes ranged from 28 to 88 patients, with a total of 246 individuals analysed. The median age across studies was 59 years, and females represented the majority (74.8% [119/159]) where sex distribution was reported. Inclusion criteria generally comprised biopsy‐proven or clinically staged ASCC with MRI and PET/CT performed for baseline staging. Reported exclusion criteria varied: prior pelvic radiotherapy, second malignancies, pelvic inflammatory disease or excessive MRI–PET interval (> 6–8 weeks). One study (Engledow [19]) excluded patients with non‐SCC histology and those under 30 years of age. See Table 1 for study characteristics.

**TABLE 1:** Study characteristics.

Author (Year)	Country	Study design	Centre type	Enrolment period	*N* (enrolled/analysed)	Age (median/mean)	Sex (F:M)	Inclusion criteria	Exclusion criteria
Bhuva (2012)	UK	Retrospective	Single centre	2009–2010	88/43	—	—	Radical treatment for anal cancer (2009–2010)	—
Di Carlo (2021)	Italy	Retrospective	Single centre	2012–2020	37/37	Median 55 (40–88)	28F: 9M	SCCAC staged with EUS, MRI, CT, PET/CT (stage I–IV)	—
Engledow (2011)	UK	Prospective	Multicentre	2005–2008	40/40	Median 57 (38–87)	25F: 15M	Histological SCC, informed consent provided	Melanoma, adenocarcinoma, age < 30 years
Horvat (2024)	USA	Retrospective	Single centre	2010–2020	28/28	Median 62 (31–78)	22F: 6M	Biopsy‐proven ASCC with MRI, CT, PET/CT	> 3 weeks imaging gap, prior treatment, poor image quality
Manafi‐Farid (2020)	Austria	Retrospective	Single centre	2008–2017	54/54	Mean 61 ± 10.6	44F: 10M	Pre‐treatment PET/CT, received therapy (2008–2017)	Prior pelvic RT, second malignancy, pelvic inflammation, > 8 weeks MRI–PET gap
Wells (2012)	UK	Retrospective	Multicentre	2008–2011	44/44 (48 scans analysed)	—	—	Anal cancer patients with PET/CT in Southwest England, MRI ≤ 6 weeks	MRI > 6 weeks

Abbreviations: ASCC, anal squamous cell carcinoma; CT, computed tomography; EUS, endoscopic ultrasound; F, female; M, male; MRI, magnetic resonance imaging; PET/CT, positron emission tomography–computed tomography; RT, radiotherapy; SCC, squamous cell carcinoma; SCCAC, squamous cell carcinoma of the anal canal.

### 3.3 Imaging Protocols and Baseline Staging

Five of the six studies reported that patients underwent combined pelvic MRI and CT chest–abdomen–pelvis (CT CAP) prior to FDG‐PET/CT [4, 5, 18, 19, 20]. In four studies (Di Carlo, Engledow, Horvat and Wells) [4, 5, 19, 20], changes in TNM stage were explicitly defined as occurring after MRI and CT CAP when PET/CT was added. In the study by Bhuva et al. [18], although all patients underwent MRI and CT CAP before PET/CT, the reporting of staging outcomes compared MRI and PET/CT directly, without clearly specifying the contribution of CT CAP to baseline staging. One study did not explicitly state the routine use of CT CAP [6].

Reporting of imaging protocols varied considerably across studies. MRI protocols were frequently underreported, with only Manafi‐Farid [6] providing detailed sequences (pelvic STIR, T2, DWI with b50/800 and T1 pre/post contrast), and Wells [20] describing small‐field‐of‐view T2 and large‐field‐of‐view T1 imaging. For PET/CT, fasting for ≥ 6 h and a 60‐min uptake period was standard in most studies, with administered activity ranging from 3 MBq/kg to 375 MBq. Reported scanners included GE Discovery ST/690/LS/710 and Philips Gemini. The MRI–PET interval was most commonly within 1 week (Di Carlo [5], Engledow [19]). However, one study reported a mean interval of 12.5 ± 13.9 days (Manafi‐Farid [6]), and Wells [20] accepted intervals up to 6 weeks.

Baseline staging distributions were variably reported. For T stage, most cohorts included a broad spectrum from T1–T4 disease, with some also reporting early stage patients (Bhuva [18], Horvat [4], Manafi‐Farid [6]). Nodal staging also demonstrated significant heterogeneity: in Di Carlo [5], 92% of patients (34/37) were node‐positive at baseline, whereas other studies reported a broader spread of nodal categories. Wells [20] did not report baseline staging distributions. A detailed breakdown of imaging protocols and TNM staging across studies is provided in Table 2.

**TABLE 2:** Study protocols and staging.

Author (Year)	MRI protocol	PET/CT protocol	MRI–PET interval	Tumour stage (T)	Nodal stage (N)
Bhuva (2012)	—	Fasting 6 h; 4.5 MBq/kg; 60‐min uptake; GE Discovery ST; non‐contrast CT; ~30 min acquisition	—	MRI: T0 = 2, T1 = 2, T2 = 16, T3 = 10, T4 = 9; PET: 3 discordances	MRI: N0 = 12, N1 = 7, N2 = 16, N3 = 5; PET discordance in 14
Di Carlo (2021)	—	Fasting 6 h; glucose < 200; 3 MBq/kg; 60 ± 5 min uptake; GE Discovery 690; 3 min/bed	≤ 1 week	Locally advanced; 34/37 LN positive	34/37 LN positive
Engledow (2011)	—	375 MBq; 60‐min uptake; GE PET/CT (64‐detector)	Median < 1 week	MRI: T1 = 5, T2 = 16, T3 = 15, T4 = 4	MRI: Nx = 0, N0 = 36, N2 = 4
Horvat (2024)	—	—	—	MRI: T0 = 3, T1 = 3, T2 = 8, T3 = 5, T4 = 9; PET: T0 = 1, T1 = 2, T2 = 14, T3 = 10, T4 = 1	MRI: N0 = 7, N1a = 15, N1b = 1, N1c = 5; PET: N0 = 11, N1a = 12, N1b = 0, N1c = 5
Manafi‐Farid (2020)	Pelvic STIR, T2, DWI (b50/800), T1 pre/post contrast	GE Discovery LS/710; standard FDG protocol	Mean 12.5 ± 13.9 days	MRI: T0 = 5, T1 = 8, T2 = 20, T3 = 6, T4 = 15; PET: T0 = 5, T1 = 8, T2 = 22, T3 = 6, T4 = 13	MRI: N0 = 28, N1a = 23, N1c = 3; PET: N0 = 28, N1a = 18, N1b = 1, N1c = 7
Wells (2012)	MRI ≤ 6 weeks; small FoV T2 + large FoV T1	Philips Gemini; fasting 6 h; catheter; bowel prep; non‐contrast CT	≤ 6 weeks	—	—

Abbreviations: CT, computed tomography; DWI, diffusion‐weighted imaging; FDG, fluorodeoxyglucose; FoV, field of view; GE, General Electric; LN, lymph node; MBq, megabecquerel; MRI, magnetic resonance imaging; N, nodal stage; PET, positron emission tomography; PET/CT, positron emission tomography–computed tomography; STIR, short tau inversion recovery; T, tumour stage.

### 3.4 TNM Staging

Across five studies (*n* = 222), FDG‐PET/CT altered staging in 22.5% (95% CI: 12.3–34.7; *I* ^2^ = 76.7%) of patients. Upstaging was more common than downstaging (16.2% [95% CI: 10.7–22.5; *I* ^2^ = 33.9%] vs. 6.3% [95% CI: 1.5–14.2; *I* ^2^ = 75.7%]), with moderate‐to‐high between‐study heterogeneity. Among upstaged patients, changes were predominantly nodal (74.6%, 95% CI: 63.2–83.1), with metastatic discovery (19.5%, 95% CI: 11.3–31.3) and T‐category revision (5.9%, 95% CI: 2.0–13.4) contributing less frequently. Analysis of staging changes by TNM axis (Table 3) demonstrated that PET/CT most frequently influenced nodal status (69.0%, 95% CI: 56.0–80.1), followed by primary tumour category (18.0%, 95% CI: 10.5–27.9) and metastatic disease (14.0%, 95% CI: 7.1–24.8). Upstaging (*n* = 35) was mainly nodal (74.6%, 95% CI: 63.2–83.1), with metastatic disease discovered in 19.5% (95% CI: 11.3–31.3) of cases. Downstaging (*n* = 16) involved tumour or nodal reassessment, but no reversals from M1 to M0 were reported. Overall, PET/CT identified previously occult metastatic disease in approximately 3.0% (95% CI: 1.1–6.9) of all patients, with potential to shift management intent from curative to palliative.

**TABLE 3:** Staging information.

Author (Year)	Upstaged	T	N	M	Downstaged	T	N	M	Overall staging change
Bhuva (2012)	12/43 (27.9%)	2/12 (16.7%)	8/12 (66.7%)	2/12 (16.7%)	5/43 (11.6%)	1/6 (16.7%)	5/6 (83.3%)	0/6 (0%)	17/43 (39.5%)
Di Carlo (2021)	5/37 (13.5%)	0/5 (0%)	4/5 (80%)	1/5 (20%)	0/37 (0%)	0/0 (−)	0/0 (−)	0/0 (−)	5/37 (13.5%)
Engledow (2011)	5/40 (12.5%)	0/5 (0%)	4/5 (80%)	1/5 (20%)	0/40 (0%)	—	—	—	5/40 (12.5%)
Horvat (2024)	—	—	—	—	—	—	—	—	—
Manafi‐Farid (2020)	5/54 (9.3%)	0/5 (0%)	4/5 (80%)	1/5 (20%)	2/54 (3.7%)	0/2 (0%)	2/2 (100%)	0/2 (0%)	7/54 (13.0%)
Wells (2012)	8/48 (16.7%)	0/8 (0%)	6/8 (75%)	2/8 (25%)	9/48 (18.8%)	6/9 (66.7%)	2/9 (22.2%)	1/9 (11.1%)	17/48 (35.4%)

Abbreviations: M, metastasis; N, nodal stage; PET/CT, positron emission tomography–computed tomography; T, tumour stage.

### 3.5 Management Change

Across six included studies, the pooled rate of management change attributable to FDG‐PET/CT following MRI was 20.7% (95% CI: 14.9–27.4; *I* ^2^ = 68.2%), though estimates varied considerably between studies (range: 0%–38%). Radiotherapy modifications accounted for the overwhelming majority of changes (18.5%, 95% CI: 12.8–25.5; *I* ^2^ = 61.7%), most often through field expansion or dose escalation to incorporate PET‐detected nodal disease. Importantly, PET also occasionally supported treatment de‐escalation by excluding false positive findings on conventional imaging. By contrast, changes in surgical planning (0.5%, 95% CI: 0.1–2.4; *I* ^2^ = 0%), chemotherapy regimens (0.9%, 95% CI 0.2–3.3; I^2^ = 0%) or transition to palliative intent (1.4%, 95% CI: 0.5–3.8; *I* ^2^ = 0%) were rare, each occurring in ≤ 2% of patients. Between‐study heterogeneity was moderate, primarily driven by the divergent findings of Bhuva (2012, 0%) and Di Carlo (2021, 38%). Excluding these outliers, the pooled management‐change rate stabilised around 15.0%, underscoring that the principal clinical value of PET/CT in this context lies in refining radiotherapy planning rather than altering systemic or surgical strategies. See Table 4 for management change resulting from PET.

**TABLE 4:** Management change resulting from PET.

Author (Year)	Total with management change (%)	RT change	Surgical change	Chemotherapy change	Palliative intent	Notes
Bhuva (2012)	0/43 (0%)	0	0	0	0	CRT protocol was fixed and independent from findings; PET altered T/N but did not change management
Di Carlo (2021)	14/37 (38%)	14	0	0	0	RT dose escalation to 50–54 Gy; CTV expansion based on PET nodal findings
Engledow (2011)	5/40 (12.5%)	4	1	0	0	RT boost to identified LN; RT field expanded to para‐aortic region; one lung resection for isolated lung metastasis
Horvat (2024)	—	—	—	—	—	PET detected additional disease, but no management changes
Manafi‐Farid (2020)	13/54 (24.1%)	12	0	0	1	RT modifications: 4 ↑ RT field to include inguinal LN; 4 ↑ RT field + dose (inguinal + iliac LN); 2 ↑ RT dose to iliac LN; 2 ↓ RT field to inguinal region; 1 patient shifted to palliative CRT
Wells (2012)	14/48 (29%)	11	0	2	1	PET findings led to: 1 liver met reclassified (multiple, systemic intent); 3 new distant mets (RT field ↑); 1 regional LN (RT field/dose ↑); 1 distant + regional LN (RT ↑); 3 suspicious on CI but PET‐negative (RT field ↓); 1 liver met on CI but PET‐negative with new LN (RT field altered)

Abbreviations: CRT, chemoradiotherapy; CT, computed tomography; CTV, clinical target volume; Gy, grey; LN, lymph node; PET, positron emission tomography; PET/CT, positron emission tomography–computed tomography; RT, radiotherapy.

### 3.6 Risk of Bias

One study was ‘very good’, four studies were ‘good’, one study was ‘satisfactory’ and zero studies were ‘unsatisfactory’. Appendix S1 summarises the results of our risk of bias assessment.

## 4 Discussion

This systematic review and meta‐analysis assessed the diagnostic and therapeutic value of FDG‐PET/CT after MRI staging in ASCC. In clinical practice, MRI remains the gold standard for local staging because of its superior soft‐tissue contrast and ability to evaluate sphincter and mesorectal involvement. Nevertheless, FDG‐PET/CT provides metabolic and whole body assessment, capable of detecting nodal or distant disease that MRI might miss. By focusing on the specific contributions of PET/CT after MRI, rather than directly comparing the two modalities, this analysis highlights the true added value of PET/CT in modern staging. Across the six studies, PET/CT modified the TNM stage in 22.5% of cases and resulted in management changes in 20.7%. Upstaging was more common (16.2% vs. 6.3% for downstaging), with most changes involving nodal disease, leading to radiotherapy field or dose adjustments in about 20% of patients. These findings confirm that even after thorough MRI staging, PET/CT offers additional clinically important information, mainly through better nodal mapping and detection of otherwise hidden metastatic disease.

Previous meta‐analyses have primarily compared PET/CT with conventional imaging (CT +/− MRI), often collating the baseline imaging reference. Mahmud et al. [8] reported pooled staging and management change rates of 21% and 18%, respectively, closely matching our findings. However, MRI was not consistently performed in all included studies in that review, possibly overestimating PET's benefit relative to modern MRI‐based staging. By contrast, our study quantifies PET/CT's residual value after MRI, providing an updated, clinically realistic estimate of its contribution to current workflows. Similarly, earlier reviews by Agarwal et al. [22] demonstrated that PET/CT detects additional nodal disease in 10%–20% of patients compared with CT alone. Our results corroborate these findings in the MRI era: approximately three‐quarters of PET‐driven upstaging involved nodal disease. Single‐centre studies by Engledow et al. [19] and Wells et al. [20] likewise reported PET‐detected inguinal and pelvic nodes that were either equivocal or normal on MRI, prompting RT modifications. Thus, the magnitude of incremental benefit observed here reflects the persistent diagnostic advantage of PET/CT despite advances in MRI quality.

MRI provides accurate delineation of local tumour extent, sphincter involvement and circumferential margins but remains limited in detecting small or metabolically active lymph nodes. Morphologic criteria alone (size > 10 mm, irregular borders, internal heterogeneity) are imperfect markers of metastatic involvement. FDG‐PET detects increased glycolytic activity and thus identifies sub‐centimetric or morphologically normal nodes that harbour disease [5, 6]. Moreover, the MRI field of view is typically confined to the pelvis, whereas PET encompasses para‐aortic and distant nodal chains. This explains why most upstaging in our analysis was nodal and why radiotherapy planning was most affected. PET also contributed to selective downstaging, usually by demonstrating metabolic inactivity in MRI‐suspicious nodes or tissue, thereby avoiding overtreatment. Although no conversions from M1 to M0 were reported, the ability to rule out false positive MRI findings supports individualised treatment planning and may reduce unnecessary toxicity.

The pooled management change rate of 20.7% highlights PET's influence on real world decision‐making. Radiotherapy modification, primarily field expansion or dose escalation to incorporate PET‐positive nodes, accounted for 18.5% of changes. These results parallel those of Mahmud et al. [8] and Adusumilli et al. [10], in which radiotherapy accounted for > 85% of PET‐driven management alterations. Prospective studies similarly show that PET‐based planning alters RT target volumes in 25%–30% of cases [6, 23], thereby directly affecting dose distribution and potentially locoregional control.

Although staging and management outcomes are linked, not all staging revisions translate into clinical action. Some nodal upstaging may occur within fields already planned for irradiation, while limited metastases might not alter curative intent. Nonetheless, the close numerical correspondence between our pooled staging (22.5%) and management change (20.7%) rates implies that most staging shifts carry genuine therapeutic consequences, reinforcing PET/CT's high practical value.

Between‐study heterogeneity was moderate to high (*I* ^2^ = 76%), likely reflecting differences in study design, patient selection, imaging acquisition protocols and variability in the interval between MRI and FDG‐PET/CT. This heterogeneity reduces precision around pooled effect estimates and warrants cautious interpretation, although the direction of effect was broadly consistent across studies. Sample sizes were modest (28–88 patients), and most series were retrospective. MRI protocols were inconsistently reported; for example, only Manafi‐Farid et al. [6] detailed comprehensive sequences (T2, STIR, DWI), limiting reproducibility. The MRI–PET interval ranged from 1 day to 6 weeks, potentially allowing true disease progression between scans. PET acquisition protocols also differed in scanner generation and uptake time. Most studies relied on composite reference standards rather than histopathology for nodal verification, risking incorporation bias. Despite these limitations, effect sizes were consistent across cohorts, supporting the robustness of pooled estimates.

This review's key strength is its targeted focus on PET's incremental contribution after MRI, filling an evidence gap left by prior comparative reviews [8]. Because MRI is now the universal baseline imaging for ASCC, quantifying PET's residual value is clinically pertinent. Moreover, by combining diagnostic and therapeutic endpoints, our study translates imaging findings into actionable clinical terms, showing that one in five patients experiences a meaningful change in treatment after PET/CT. These results substantiate current ESMO [24] and NCCN [25] recommendations that endorse PET/CT for baseline staging, particularly in locally advanced or equivocal cases. Our quantitative synthesis provides contemporary, evidence‐based justification for that guidance.

Despite over a decade of research, important gaps remain. Our reviews were entirely retrospective, showing the need for prospective review. There was variability in imaging acquisition protocols across studies. MRI techniques were inconsistently reported, with differences in field of view and variable use of diffusion‐weighted imaging, factors known to influence sensitivity for small or borderline lymph nodes. Similarly, PET/CT parameters such as uptake time, administered activity and scanner generation varied between studies and may affect detection of low‐volume nodal or distant disease. These technical differences likely contribute to the heterogeneity observed in pooled staging and management outcomes and should be considered when interpreting the magnitude of PET/CT's incremental benefit. Future multicentre studies should standardise MRI and PET protocols, including field‐of‐view, DWI parameters and uptake times. Furthermore, it remains unproven whether PET‐driven RT adaptations improve survival or locoregional control; this warrants prospective validation [19, 22]. Further review into specific markers, such as SUVmax, metabolic tumour volume and total lesion glycolysis, may offer insight on prognosis and should be incorporated into future studies. A further limitation of this analysis relates to heterogeneity in reporting of baseline systemic imaging, particularly the incorporation of CT CAP into staging comparisons. Although the majority of included studies reported combined MRI and CT CAP staging prior to FDG‐PET/CT, one study did not explicitly document routine CT CAP use, and another compared MRI and PET/CT directly despite prior CT CAP imaging. This variability may modestly overestimate PET‐related upstaging compared with fully standardised MRI and CT CAP pathways. Nevertheless, because most PET‐driven staging changes in our pooled analysis were nodal rather than distant metastasis, the principal findings regarding PET/CT's incremental value for nodal assessment and radiotherapy planning remain robust. The interval between MRI and FDG‐PET/CT varied across studies, ranging from < 1 week to up to 6 weeks. This raises the possibility that some PET‐related upstaging may reflect interval disease progression rather than improved detection alone and should be considered when interpreting pooled staging estimates. Nonetheless, the directionally consistent results across several years of literature affirm that PET/CT meaningfully impacts MRI‐based staging, primarily through improved nodal assessment and radiotherapy planning.

## 5 Conclusion

FDG‐PET/CT performed after MRI staging in ASCC changes TNM stage in roughly one in five patients and alters clinical management, predominantly radiotherapy planning, in a similar proportion. The incremental benefit lies chiefly in the detection and characterisation of nodal disease and, to a lesser degree, occult metastases. These findings confirm and refine previous meta‐analyses by quantifying PET's residual value once MRI has defined local extent. PET/CT remains an essential adjunct to MRI, enhancing confidence and precision in staging and treatment planning. Future prospective and PET/MRI studies should assess whether these imaging‐driven adaptations translate into improved oncologic outcomes.

## Author Contributions

H.C.T. was responsible for the conceptualisation of the study, conducting the literature search, data extraction, methodology design, data analysis and drafting of the manuscript. J.M.B. contributed to data extraction, interpretation of findings and critical revision of the manuscript. M.E.K. provided supervision, contributed to the interpretation of results and critically revised the manuscript. B.M.M.C. contributed to study oversight, interpretation of results and critical revision. All authors reviewed and approved the final version of the manuscript.

## Funding

The authors have nothing to report.

## Disclosure

Permission to reproduce material from other sources: No copyrighted material, figures or tables requiring permission for reproduction have been used in this article.

## Ethics Statement

Ethics approval was not required for this study, as it is a systematic review and meta‐analysis of previously published literature and does not involve human participants, identifiable data or animal research.

## Consent

The authors have nothing to report.

## Conflicts of Interest

The authors declare no conflicts of interest.

## Supporting information


**Appendix S1:** ara70071‐sup‐0001‐AppendixS1.docx.

## Data Availability

All data generated or analysed during this study are included in this published article and its Supporting Information. No new, individual patient data were collected or generated.
