# Supplementary material for: Diagnostic and Therapeutic Impact of FDG‐PET/CT Following MRI Staging in Anal Cancer: A Systematic Review and Meta‐Analysis
Source: J Med Imaging Radiat Oncol. 2026 Jan 19;70(2):188–97. doi: 10.1111/1754-9485.70071 (PMC12954381; doi:10.1111/1754-9485.70071)
Supplement: Supplementary file 1 — Appendix S1: ara70071‐sup‐0001‐AppendixS1.docx. [file ARA-70-188-s001.docx]

| Author | **Selection** | | | | **Comparability.** | **Outcome** | | | **Quality** |
| --- | --- | --- | --- | --- | --- | --- | --- | --- | --- |
|  | **Representativeness of the exposed cohort** | **Sample size (<20 = no star)** | **Open cases only included** | **Ascertainment of the exposure** | **The subjects in different outcome groups are comparable** | **Assessment of outcome** | **Less than 10% missing data?** | **Average Follow up period (> 12 months)** |  |
| Bhuva (2012) | **🟑** | **🟑** | **🟑** | **🟑** | / | **🟑** | **🟑** | / | 6 |
| Di Carlo (2021) | **🟑** | / | **🟑** | / | **🟑** | **🟑** | **🟑** | **🟑** | 6 |
| Engledow (2011) | **🟑** | **🟑** | **🟑** | **🟑** | **🟑** | **🟑** | / | / | 6 |
| Horvat (2024) | **🟑** | / | / | **🟑** | **🟑** | / | **🟑** | / | 4 |
| Manafi-Farid (2020) | **🟑** | **🟑** | **🟑** | **🟑** | **🟑** | **🟑** | **🟑** | **🟑** | 8 |
| Wells (2012) | **🟑** | /** ** | **🟑** | **🟑** | **🟑** | **🟑** | / | **🟑** | 6 |

**Supplementary Appendix 1: Risk of bias assessment (Newcastle-Ottawa scale)**
